# The Indications and Contra-Indications for Curetting the Uterus

**Published:** 1908-06

**Authors:** J. Inglis Parsons

**Affiliations:** Surgeon, Chelsea Hospital for Women


### The Indications and Contra-Indications for Curetting the Uterus.

By J. INGLIS PARSONS, M. D., M.R.C.P.,
Surgeon, Chelsea Hospital for Women.

[*The Hospital, May 2, 1908.*]

THE most common symptoms for which curetting is done are Menorrhagia and Metrorrhagia. In order to succeed it is necessary to form a correct diagnosis of the cause. This cannot always be done until the patient is under an anesthetic and the uterus is dilated and explored. Therefore, in all cases one should be chary of promising a successful result.

The most obvious indication for the operation is afforded by a history of recent parturition or miscarriage followed by a persistent or irregular red discharge. Here we may, with few exceptions, advise the operation with confidence and give a good prognosis. The exceptions are these. The patient may be suffering from chorion epithelioma, or the miscarriage may have been due to the presence of a small fibroid in the wall of the uterus. In the former case, curetting will, if anything, rather increase the metrorrhagia. If a fibroid is present the operation may stop the menorrhagia for a short time, but the loss soon recurs.

Another point is this, that patients after miscarriage frequently have menorrhagia for two or three months even when the uterus is quite empty. If the loss is caused by retention of any of the products of conception a vaginal examination invariably reveals a larger opening of the external os than is normal, and during the operation the uterus is found to dilate quite easily. Even if nothing is found after dilatation, curetting appears to stimulate the uterus to contract and undergo involution.

In these cases great care is required in the use of the curet. The walls of the uterus are often very flabby and softened; any roughness may result in the curet penetrating the wall into the peritoneal cavity. On this account it is better to use the finger, if possible In many cases the finger will not reach the upper end of the uterus. Ovum forceps may be tried, but the curet has to be relied on in the end.

In dealing with septic cases it is most important to remember that the microörganisms are generally confined to the uterine cavity at first, and that the walls of the uterus with dilated vessels and innumerable phagocytes form a protecting barrier to the invasion of the system. Any cases, therefore, with retained products, and pyrexia, must be dealt with somewhat differently and the use of the curet avoided if possible. The dilatation should be done very gradually, so as to avoid any risk of splitting the endometrium. Before doing anything else, the cavity of the uterus should be thoroughly irrigated with an antiseptic solution. The temperature of this should not be more than 98° F. or it will cause the uterus to contract. I generally use chinosol, because it is non-toxic and a powerful antiseptic.

The next step is to remove the offending fragment, with the finger if possible. Should it be found necessary to use the curet, a blunt one should be chosen. The operator must not attempt to scrape the whole uterine cavity, because he would by that means open up a large surface by which the septic organisms could gain entrance to the body. By going over the cavity very gently with the blunt curet he will be able to feel it pass over a projection and locate its position without injuring the protective layer in the rest of the endometrium. When the fragments have been removed, a second douching should follow and finally the cavity should be gently swabbed with tincture of iodine.

For the menorrhagia produced by fibromyoma, curetting is not often required now that the mortality from hysterectomy is so low. The exceptions are the patients who refuse the major operation and those who have only one or two small tumors without any severe symptoms beyond the increased loss. If the menorrhagia can be stopped in the latter class, they are in a great measure restored to health and still capable of becoming mothers. Hysterectomy for these cases is unjustifiable until other treatment has had a fair trial. The benefit derived from curetting for fibromyoma rarely lasts more than a few months. In some instances the distortion of the uterine canal makes it impossible to carry out the operation efficiently. In any case, the results of curetting as a hemostatic in these patients is not to be compared with Apostoli's treatment. The latter will, in eight cases out of ten, arrest the hemorrhage for three to four years and also considerably check the growth of the tumor.

The diagnosis of the cause of the menorrhagia in the two groups already discussed is fairly easy, but when there are no physical signs of carcinoma, myoma, or polypus, and no history whatever of parturition or miscarriage for some years, the problem is more difficult.

In this class there are more causes to account for the hemorrhage and more difficulty in defining it. If, on examination, we find the uterus is not enlarged or not more than we might expect in a woman who has had one or two children, we should give a guarded opinion and state openly that a dilatation is necessary before a definite opinion can be formed. The causes in this group may be adenoma of the endometrium, fibrosis of the uterine walls, carcinoma, or retroflexion. To these we might add polypus uteri, but the latter usually causes some metrorrhagia, as does carcinoma also.

Adenoma of the uterus causes thickening and increased vascularity of the endometrium. The condition varies through wide limits. There may be only a slight proliferation of the uterine glands, or we may find small masses of growth distinguishable only by the microscope from carcinoma.

When the endometrium is uniformly thickened, and only to a moderate extent, the prognosis is good and curetting will probably cure the patient. If, however, there are small masses of growth somewhat resembling carcinoma, the adenoma is likely to recur, although the patient benefits for a time from the removal of the vascular masses. In a case of this kind I have had finally to do a hysterectomy, although the growth was absolutely a benign adenoma with no sign of carcinoma.

In striking contrast to adenoma of the uterus is the thin endometrium found in fibrosis. Many of the muscular fibres are replaced by fibrous tissue, so that a rasping sensation and sound are conveyed to the operator as the curet passes over the walls of the uterus. A guarded prognosis should always be given. In a few of these patients improvement may follow for a short time, in many there is none at all, and in some the menorrhagia is actually increased by the operation. As a rule, hysterectomy has to be done for hospital patients, to arrest the loss and progressive anemia. Apostoli's treatment is often successful and is suitable to private patients, who can afford the time necessary for this method of treatment, and who often have objections to removal of the uterus.

Retroversion or retroflexion is a not unfrequent cause. Why some of these patients should have menorrhagia and not others probably depends on the size of the pelvis and other factors not within the scope of this paper. The mistake is often made of curetting the uterus without correcting the displacement. With a history of symptoms dating back not more than a few months, it is only necessary to replace the uterus and keep it up. The menorrhagia and leucorrhea will then cease without further treatment. If, as is often done, the uterus is curetted and the displacement left, the symptoms go on just the same, to the disgust of the patient and the humiliation of the operator.

It is only in old-standing cases, where congestion produced by the retroflexion has been active for some time, that it is necessary to curet. Even then the displacement should be corrected first and the curetting done afterwards, if necessary.

Diseased appendages very often cause severe menorrhagia, more particularly when the ovary on either side is diseased. Considerable experience is necessary sometimes to detect the disease by a bimanual examination, when there is not much enlargement of the ovary. The best plan is to examine under an anesthetic, prepared to curet if nothing is found, or to open the abdomen and remove the appendage if it is felt to be enlarged and diseased.

Leucorrhea is more often than not a symptom. Prolapse, retroflexion, polypus, etc., will always cause it. Any of the various conditions that lower the general health will cause leucorrhea. But over and beyond these cases there are some women, otherwise quite healthy, who suffer from continued leucorrhea. Just as others may be quite well except for a nasal catarrh, a delicate throat, an impaired digestion, or chronic constipation, so do these patients appear to have a weak spot in their system in the endometrium. Curetting will generally do good by removing an unhealthy membrane, while the dilatation and scraping stimulate the uterus to form a new and healthy endometrium.

If, on the other hand, the discharge is yellow, it means infection of the endometrium by some microörganism, probably the gonococcus. Under these circumstances curetting does very little good, if any. It is better to dilate and irrigate the uterine cavity thoroughly with a non-irritating antiseptic, such as chinosol, and then swab the uterus with tincture of iodine. The latter application is then repeated every other day. In very obstinate cases I have found the constant current more efficacious than anything else; probably because it stimulates the uterus and increases its resisting power to the invasion of microörganisms.

Anteflexion of the uterus associated with dysmenorrhea and sterility causes thickening of the endometrium with proliferation of the glands resembling adenoma. Curetting is of great value to these patients, and may cure the sterility. The dilatation must be done slowly and carefully, for if the walls of the uterus are injured, the dysmenorrhea may be worse than before.

For inoperable carcinoma of the cervix the operation is most useful. The ulceration should be thoroughly and carefully scraped, and then mopped over with formalin or liquid carbolic. The foul discharge and hemorrhage are usually stopped for some months, while the improvement in the patient's health and comfort are much greater than would be expected from so simple a procedure.

